# High Dose Ascorbate Causes Both Genotoxic and Metabolic Stress in Glioma Cells

**DOI:** 10.3390/antiox6030058

**Published:** 2017-07-22

**Authors:** Maria Leticia Castro, Georgia M. Carson, Melanie J. McConnell, Patries M. Herst

**Affiliations:** 1 School of Biological Sciences, Victoria University, P.O.Box 600, Wellington 6140, New Zealand; leticia.castro@vuw.ac.nz (M.L.C.); georgia.carson@hotmail.co.nz (G.M.C.); Melanie.McConnell@vuw.ac.nz (M.J.M.); 2 Malaghan Institute of Medical Research, P.O.Box 7060, Wellington 6242, New Zealand; 3 Department of Radiation Therapy, University of Otago, P.O.Box 7343, Wellington 6242, New Zealand

**Keywords:** high dose ascorbate, H_2_O_2_, radiation, oxidative stress, genotoxic stress, metabolic stress, DNA synthesis arrest

## Abstract

We have previously shown that exposure to high dose ascorbate causes double stranded breaks (DSBs) and a build-up in S-phase in glioblastoma (GBM) cell lines. Here we investigated whether or not this was due to genotoxic stress as well as metabolic stress generated by exposure to high dose ascorbate, radiation, ascorbate plus radiation and H_2_O_2_ in established and primary GBM cell lines. Genotoxic stress was measured as phosphorylation of the variant histone protein, H2AX, 8-oxo-7,8-dihydroguanine (8OH-dG) positive cells and cells with comet tails. Metabolic stress was measured as a decrease in NADH flux, mitochondrial membrane potential (by CMXRos), ATP levels (by ATP luminescence) and mitochondrial superoxide production (by mitoSOX). High dose ascorbate, ascorbate plus radiation, and H_2_O_2_ treatments induced both genotoxic and metabolic stress. Exposure to high dose ascorbate blocked DNA synthesis in both DNA damaged and undamaged cell of ascorbate sensitive GBM cell lines. H_2_O_2_ treatment blocked DNA synthesis in all cell lines with and without DNA damage. DNA synthesis arrest in cells with damaged DNA is likely due to both genotoxic and metabolic stress. However, arrest in DNA synthesis in cells with undamaged DNA is likely due to oxidative damage to components of the mitochondrial energy metabolism pathway.

## 1. Introduction

The last decade has seen a renewed interest in intravenous high dose (pharmacological) ascorbate (AA) as an anticancer treatment. Most authors in the field attribute the anticancer effect of high dose AA to its pro-oxidant effect. In the extracellular acidic and metal-rich tumour environment, high dose AA generates extracellular hydrogen peroxide (H_2_O_2_), which diffuses into adjacent cancer cells and overwhelms the anti-oxidant defence system (reviewed by [1]). The resulting oxidative stress damages macromolecules [1] as well as depleting NAD^+^ and ATP levels [2,3,4,5]. Yun and colleagues reported recently that the oxidised form of AA, dehydroascorbate (DHA) rather than AA was responsible for selectively killing glycolysis-driven colorectal cancer cells with BRAF and KRAS mutations [6]. DHA is transported into cells through glucose transporters (GLUT-1), where it is reduced back to AA at the expense of glutathione (GSH), causing oxidative stress and inhibition of glyceraldehyde-3-phosphate dehydrogenase (GAPDH) and thus glycolysis, leading to ATP depletion [6,7]. In addition to causing oxidative stress, AA has been shown to increase hypoxia inducible factor, HIF-1, hydroxylase activity, leading to a decrease in HIF-1 pathway activation and a less aggressive phenotype in colorectal [8] and endometrial cancer [9], and inhibit the proliferation of breast cancer MCF-7 mammospheres [10].

Most authors have reported that high dose AA has little or no effect on non-cancerous cell lines (reviewed by [11]) and few side effects in animal models [12,13] or clinical trials [4,14,15]. Cancer specificity has been attributed to the acidic and metal-rich tumour micro-environment combined with the inferior anti-oxidant capacity and compromised DNA repair pathways of tumour cells [16,17,18,19]. Combining high dose AA with ionizing radiation should therefore radio-sensitize highly radiation resistant glioblastoma (GBM) cells [20,21] and improve the dismal prognosis for GBM patients [22].

Our group has studied the effect of high dose AA on radio-sensitization of GBMs in several studies. We initially showed that a GBM cell line isolated from a GBM patient was much more sensitive to high dose AA, radiation and combined treatments than a mouse-derived normal glial cell line [23]. However, a subsequent more detailed study showed that six human GBM cell lines, a human glial cell line and human umbilical vein endothelial cells were similarly sensitive to high dose AA and/or radiation. Sensitivity depended on their antioxidant and DNA repair capacity regardless of their cancerous status [24]. We further showed that exposure to high dose AA caused accumulation in S-phase as well as genotoxic stress. Genotoxic stress was demonstrated by a higher percentage of cells with foci caused by phosphorylation of the variant histone protein (H2AX) associated with the DNA damage response (γH2AX) as well as more γH2AX foci per cell [23,24]. The number of γH2AX foci correlates well with the number of double strand breaks (DSBs) generated by ionising radiation [25,26,27,28]. However, fewer than half of γH2AX foci induced by H_2_O_2_ [25,26,27] and UV [28] are associated with DSBs; with foci produced during replication likely representing stalled replication forks, which can either be repaired or progress to DSBs [25,26,27,28]. Another type of DNA lesion that causes genotoxic stress are 8-oxo-7,8-dihydroguanine (8OH-dG) lesions caused by aggressive hydroxyl free radicals generated by H_2_O_2_ in the close vicinity of DNA [18]. Although these lesions are also present in some untreated GBM cell lines and many GBM tumours [21], they are generated specifically in response to H_2_O_2_ [25,26,27]. The lesions are rapidly repaired by base excision repair (BER) and if unrepaired may generate single stranded breaks [18] or double stranded breaks [25,26,27,28]. Metabolic stress, in the form of low NAD^+^ and low ATP levels as a result of high dose AA exposure, has been shown by several authors [2,3,4,5]. In this paper, we analysed the extent of genotoxic and metabolic stress and the effect on DNA synthesis by high dose AA in established and patient-derived GBM cell lines and compared these effects to those of radiation, H_2_O_2_ and combined treatments.

## 2. Materials and Methods

### 2.1. Materials

Unless otherwise noted, tissue plasticware was purchased from Corning (In Vitro Technologies, Auckland, New Zealand); all cell culture reagents were from Gibco BRL (Thermo Fisher Scientific, Auckland, New Zealand). Alexa Fluor 488 anti-H2AX-Phosphorylated (Ser139) Antibody was from BioLegend (Norrie Biotech, Auckland, New Zealand). Rabbit anti-8-OHdG polyclonal antibody (J-1: sc-139586) was from, Santa Cruz Biotech (Dallas, Texas, USA) and isotype control (IgG/10500C) was from Thermofisher Scientific (Wellington, New Zealand). Goat Polyclonal Anti-Rabbit IgG H&L (Alexa Fluor^®^ 488) secondary antibody was from Abcam (Cambridge, MA, USA). Foxp3/Transcription Factor Fixation/Permeabilization Concentrate and Diluent was purchased from eBioscience (Huntingtree Bioscience Supplies, Auckland, New Zealand). Click-iT EdU Alexa Fluor Flow Cytometry Assay Kits, Vybrant DyeCycle Stains, MitoSOX™ Red Mitochondrial Superoxide Indicator and MitoTracker^®^ Red CMXRos were purchased from Life Technologies (Thermo Fisher Scientific, Auckland, New Zealand). CellTiter 96^®^ AQueous One Solution Cell Proliferation Assay (MTS) was sourced from Promega Corporation (Madison, WI, USA). Luminescent ATP Detection Assay Kit was obtained from Abcam (Cambridge, MA, USA). Sodium ascorbate, and all other chemicals and reagents were from Sigma Chemical Company (St. Louis, MO, USA).

### 2.2. Cell Lines

GBM cell lines, LN18, U87MG and T98G were obtained from the American Type Culture Collection. Primary GBM cells (NZG0809, NZG1003) were isolated and cultured from GBM material as previously described [29]. GBMs were grown in RPMI-1640 supplemented with 5% (*v*/*v*) FBS. All cells were maintained in a humidified incubator at 37 °C/5% CO_2_.

### 2.3. Ascorbate Treatment

Exponentially growing cells (30–40% confluent) were seeded 24 h prior to treatment in 6 well plates (3–5 × 10^4^ cells/well). Cells were exposed to 5 mM AA in media for 1 h, washed in Dulbecco’s Phosphate Buffer Saline (PBS, 1.4 M NaCl, 27 mM KCl, 170 mM NaH_2_PO_4_, 17.6 mM KH_2_PO_4_) and re-incubated in fresh medium. Cells that received radiation were irradiated in the presence of AA.

### 2.4. Radiation Treatment

Exponentially growing cells (30–40% confluent) were irradiated fully immersed in medium with 6 Gy using Cesium-137 γ-rays (Gammacell 3000 Elan, Best Theratronics, Kanata, ON, Canada). After irradiation, cells were re-incubated in fresh medium.

### 2.5. DNA Synthesis: EdU Incorporation

Fluorescent detection of incorporated thymidine analogue EdU (5-ethynyl-2’-deoxyuridine) was used as a measure of DNA synthesis. Cells were pulse-labelled with 4 µM EdU for 1 h prior to analysis. Incorporated EdU was detected using a copper catalyzed covalent reaction between Click-iT^®^ EdU Alexa Fluor^®^ 488 or Alexa Fluor^®^ 647 dye azide and an alkyne on the ethynyl moiety of EdU. Briefly, cells were pulse labelled with EdU for 1 h prior to harvesting, washed twice in FACS buffer (PBS + 1% Bovine Serum Albumin (BSA), and incubated in Click-iT fixative at room temperature for 15 min in the dark. Cells were washed and resuspended in saponin-based permeabilisation buffer for 15 min prior to incubation in the Click-iT AF647 reaction cocktail for 30 min in the dark at room temperature. For DNA content staining, these cells were incubated in a 5 µM Vybrant Dye Cycle Green/HBSS staining solution for 30 min at 37 °C prior to analysis by flow cytometry using a BD FACSCanto II (Becton Dickinson, San Jose, CA, USA). Data were analysed using FlowJo (TreeStar, Ashland, OR, USA).

### 2.6. γH2AX Labelling

Genotoxic stress was determined by measuring the extent of phosphorylation of the histone protein, H2AX, using Alexa Fluor 488 labelled anti-γH2AX antibody in permeabilised cells. EdU labelled cells were harvested and washed in PBS buffer, distributed into 96 well plates (5 × 10^5^ cells/well), washed in 200 μL FACS buffer (PBS + 1% BSA), pelleted at 400× *g* (in a Megafuge 2.0R, Heraeus centrifuge, Labcare, Buckinghamshire, UK) for 4 min and fixed in 200 μL Forkhead box P3 (FoxP3)/Transcription Factor Fixation/Permeabilization solution for 45 min at room temperature. Cells were washed twice in 200 μL 1x Permeabilization/Wash buffer (PBS, 1% BSA, 0.5% Saponin), pelleted and incubated in Click-iT^®^ AF647 reaction cocktail for 30 min in the dark. Cells were then washed twice in Perm/Wash buffer and incubated in antibody solution (50 μL anti-γH2AX antibody or isotype control diluted 1:200 in 1x Perm/Wash buffer) at 4 °C overnight. Following this, cells were washed twice in Perm/Wash buffer and resuspended in 400 μL FACS buffer. Cells were analysed by flow cytometry using a BD FACSCanto II (Becton Dickinson, San Jose, CA, USA) and FlowJo software (TreeStar, Ashland, OR, USA).

### 2.7. 8OH-dG Lesions

Harvested cells were washed in PBS buffer and distributed into 96 well plates (5 × 10^5^ cells/well), washed in 200 μL FACS buffer (PBS + 1% BSA), pelleted at 400× *g* (in a Megafuge 2.0R, Heraeus centrifuge, Labcare, Buckinghamshire, UK) for 4 min and fixed in 200 μL Foxp3/Transcription Factor Fix/ Perm solution for 45 min at room temperature. Cells were washed twice in 200 μL 1x Permeabilization/Wash buffer (PBS, 1% BSA, 0.5% Saponin), pelleted and resuspended in primary antibody solution (50 μL rabbit anti-8OH-dG polyclonal antibody or isotype control (diluted at 400 ng/mL in 1x Perm/Wash buffer) at 4 °C overnight, washed twice in Perm/Wash buffer and resuspended in secondary antibody solution (Goat Polyclonal Anti-Rabbit IgG H&L (Alexa Fluor^®^ 488), Abcam) at 1000 ng/mL) for an hour at room temperature. Cells were then washed twice in Perm/wash buffer and resuspended in 400 μL FACS buffer for analysis by flow cytometry using a BD FACSCanto II (Becton Dickinson, San Jose, CA, USA) and FlowJo software (TreeStar, Ashland, OR, USA).

### 2.8. Comet Tail Assay

Glass slides (LabServ Superfrost Plus) were pre-coated with 1% normal melting point agarose (Invitrogen UltraPure Agarose) and air-dried for 24 h. Cells were added to 1% low melting point agarose at a ratio of 1:10 (v/v) to final cell concentration 1 × 10^4^ cells/mL, and dropped onto agarose coated slides. Agarose and cells were air dried for 30 min at room temperature, then lysed in pre-chilled lysis solution (2.5 M NaCl, 100 mM EDTA pH 10, 10 mM Trizma, 1% sodium lauryl sarcosinate, and 1% Triton X-100, pH 10) for 1 h at 4 °C. Slides were rinsed in 1x Tris-Borate-EDTA buffer TBE, and equilibrated for 30 min in 1x TBE before electrophoresis at 30 volts/cm for 60 min. Slides were stained with 10 μg/mL propidium iodide for 20 min at 4 °C, rinsed in TBE, and imaged on a fluorescent microscope (Olympus BX51 microscope with TXRED filter). Cell nuclei were analysed using ImageJ Comet Assay plugin, based on an NIH Image Comet Assay by H.M. Miller (https://www.med.unc.edu/microscopy/resources/imagej-plugins-and-macros/comet-assay) from Robert Bagnell. Briefly, tight ovals are drawn around the head and the tail. The tail length is the distance from the centre of the head (defined as the average of XY coordinates of all pixels in the head oval) to the centre of mass of the tail (defined as the brightness-weighted average of XY coordinates of the selected tail oval). Tail length is calculated as the Pythagorean distance between the two points. ImageJ outputs were imported into Microsoft Excel and averages were imported into Prism (Graphpad) V6.0 for analysis.

### 2.9. MTS Assay

The colorimetric CellTiter 96^®^ AQueous One Solution Cell Proliferation Assay containing the soluble tetrazolium compound MTS (3-(4,5-dimethylthiazol-2-yl)-5-(3-carboxymethoxyphenyl)-2-(4-sulfophenyl)-2H-tetrazolium, inner salt) and electron coupling reagent PES (phenazine ethosulfate) were utilised for assessment of NADH flux in cells. Cells (5 × 10^3^ cells/well) were plated into 96 well plates and incubated overnight. Cells were treated and washed three times in 300 µL of PBS. 100 µL of culture media was replaced and 20 µL of CellTiter 96^®^ AQueous One Solution Reagent was added. Plates were incubated for 1−4 h at 37 °C and absorbance was measured at 490 nm on an Enspire 2300 plate reader (Perkin-Elmer, Shelton, CT, USA).

### 2.10. MitoTracker^®^ Red CMXRos

Mitochondrial membrane potential was assessed using the cell-permeant X-rosamine derived MitoTracker^®^ Red CMXRos dye. Treated cells and untreated cells were harvested and incubated in 50 nM CMXRos in PBS for 30 min at 37 °C. Cells were analysed by flow cytometry using a BD FACSCanto II (Becton Dickinson, San Jose, CA, USA) and FlowJo software (TreeStar, OR, USA).

### 2.11. MitoSOX™ Red Mitochondrial Superoxide Indicator

Live-cell permeant MitoSOX Red superoxide indicator targets mitochondria, where it is selectively oxidized by mitochondrial superoxide to exhibit red fluorescence. Treated and untreated cells were harvested and incubated in 5 µM MitoSOX in PBS for 10 min at 37 °C, fluorescent signal detected by flow cytometry using a FACSCanto II (Becton Dickinson, San Jose, CA, USA) with FlowJo software (TreeStar, OR, USA).

### 2.12. ATP Measurements

Cellular ATP was assessed using the firefly luciferase based Luminescent ATP Detection Assay Kit (Abcam, Cambridge, UK) according to manufacturer’s instruction. Cells (2 × 10^3^ cells/well) were plated into 96 well cell culture treated white plates and incubated overnight. Cells were treated, washed, and lysed in 50 µL of detergent for 5 min on an orbital shaker at 700 rpm. Substrate solution (50 µL) was added and cells were incubated in the dark for a further 5 min on an orbital shaker at 700 rpm. The plate was dark adapted for 10 min prior to luminescent reading using an Enspire plate reader (Perkin-Elmer, Shelton, CT, USA).

### 2.13. Cell Viability

GL261 cells were collected 48 h after treatment by trypsinization, washed in PBS and resuspended in 1 μg/mL propidium iodide, for cell count and dye exclusion using flow cytometry using a BD FACSCanto II (Becton Dickinson, San Jose, CA, USA) and FlowJo software (TreeStar, Ashland, OR, USA). All viability assays were completed at least three times in triplicate.

### 2.14. Statistical Analysis

Data were analysed using Excel (Microsoft v. 2010; Redmond Campus, Redmond, WA, USA) or Prism (Graphpad) V6.0. All experiments were done at least three times in triplicate; values are averages ± standard error of the means (SEM). Flow cytometry plots are representative of at least 3 separate experiments.

## 3. Results

### 3.1. GBM Cells Have Different Sensitivities to High Dose Ascorbate

**Figure 1:**
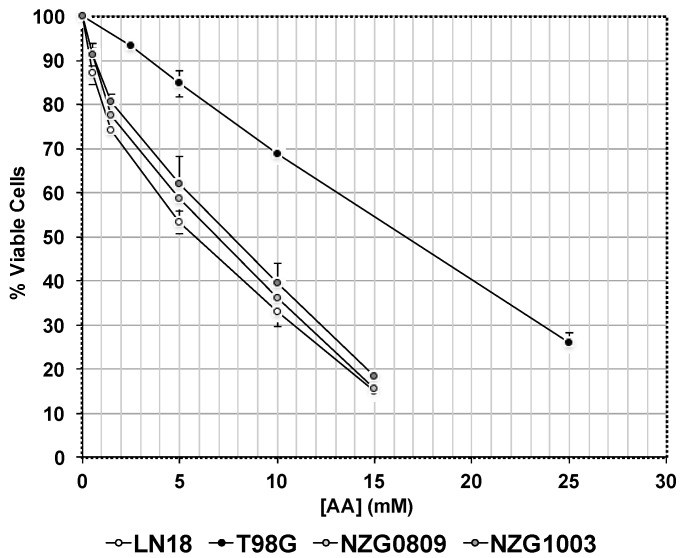
Sensitivities of different glioblastoma (GBM) cells to high dose ascorbic acid (AA). Cells were exposed to different concentrations of AA. Viability was measured by Trypan blue exclusion after 48 h. Values are averages ± standard error of the means (SEM) of at least 3 independent experiments in triplicate.

Our previous research showed that TG98G cells were less sensitive to 5 mM AA as evidenced by higher clonogenicity than other GBM cells, most likely because of its very high antioxidant content [26]. A more in-depth analysis of AA sensitivity (Figure 1) showed that the IC_50_ of T98G cells was much higher (16.5 mM AA) than that of LN18, NZG0809 and NZG1003 cells (5.8, 6.8 and 7.6 mM AA respectively).

### 3.2. High Dose Ascorbate Generates Oxidative Damage and Double-Stranded DNA Breaks

**Figure 2:**
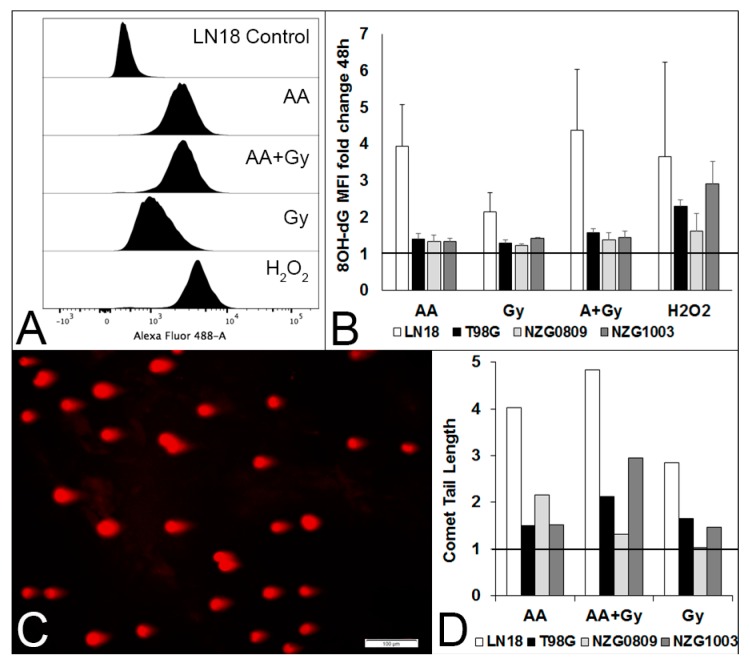
Different types of DNA damage after a 1 h exposure to 5 mM AA, 6 Gy, 5 mM AA + 6 Gy and 500 μM H_2_O_2_. (**A**) Histograms of 8OH-dG lesions 48 h after treatments of LN18 cells. (**B**) Median fluorescence intensity (MFI) fold change compared with untreated cells of LN18, T98G, NZG0809 and NZG1003 cells. Values are averages ± SEM of at least 3 independent experiments in triplicate. Increased 8OH-dG lesions for H_2_O_2_ treatment over AA, Gy and AA + Gy was statistically significant (*p* < 0.05: unpaired two-tailed student *t*-test) for T98G and NZG1003. C. Representative photograph of comet tails (DSBs) of LN18 cells after a 1 h exposure to 5 mM AA. D. Fold change compared with controls of comet tail length of the different cell lines after a 1 h exposure to 5 mM AA, 6 Gy, 5 mM AA + 6 Gy compared to untreated controls (set at 1). Average number of comet tails measured per cell line: 278 (controls), 162 (AA), 220 (Gy) and 272 (AA + Gy).

We have previously used γH2AX foci as a measure of DSBs [23,24]. However, less than half of γH2AX foci after H_2_O_2_ exposure correlate closely with DSBs [25,26,27]. As most of the high dose AA effects are thought to be mediated though H_2_O_2_ formation and H_2_O_2_ has been shown to generate 8OH-dG lesions, we determined the level of 8OH-dG lesions in our cell lines before and after exposure to 5 mM AA, 6 Gy radiation, a combination of AA and radiation and 500 μM H_2_O_2_. We saw very few lesions 1–2 h after treatments (results not shown) but increasing numbers after 48 h (Figure 2A,B). We performed single cell gel electrophoresis to confirm that high dose AA does generate some DSBs, as evidenced by the presence of “tails” in the comet tail assay (Figure 2C,D). Notably, there were cells without comet tails (and thus DSBs) after exposure to AA, consistent with previous data demonstrating the presence of γH2AX-negative cells [24].

### 3.3. High Dose Ascorbate Abrogates DNA Replication Which Does Not Resolve over Time

**Figure 3:**
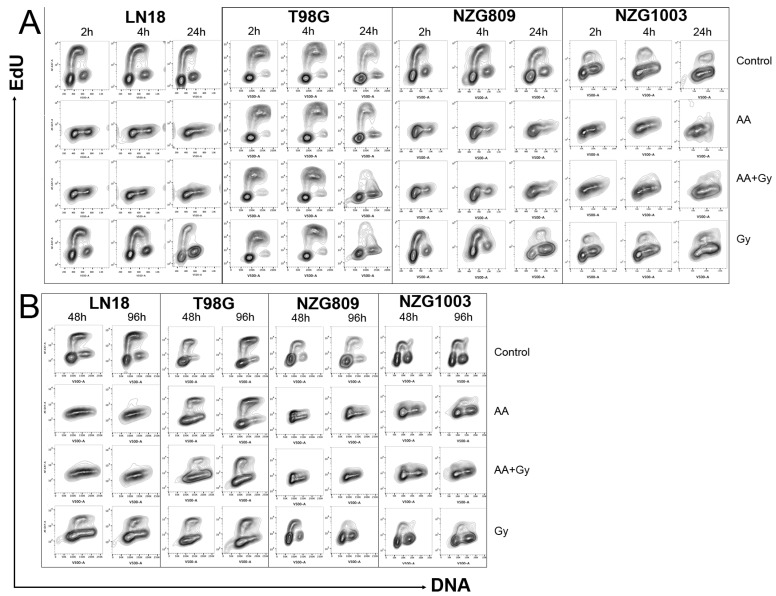
DNA synthesis indicated by EdU incorporation (*y*-axis) and DNA content by nucleic acid content (*x*-axis). G1 cells, with half the content of G2 cells are EdU negative, whereas cells undergoing synthesis have incorporated EdU into the newly synthesised DNA with subsequent increased signal. Cells were harvested at early time points (**A**) and late time points (**B**) after treatments. Flow cytometry plots are representative of at least three independent experiments.

We previously showed that GBM cells accumulate in S-phase 24 h after transient exposure to high dose AA [23,24], suggestive of replication fork collapse [25,26,27,28]. Here, we measured the extent of DNA synthesis by incorporation of the modified nucleotide ethynyl deoxyuridine (EdU) at early (Figure 3A) and late (Figure 3B) time points after treatments. Plotting EdU incorporation against DNA content resulted in a typical distribution for dividing cells, where the strongly EdU positive cells were predominantly in S-phase with an intermediate DNA content. Strikingly, a 1 h AA exposure completely arrested DNA replication within 2 h in three of the four cell types tested, which was not resolved by 96 h. DNA synthesis in T98G was much less affected by AA, consistent with previous data suggesting this cell line is less sensitive to AA (Figure 1, [24]. In most cells, radiation resulted in a G2 arrest by 24 h with a resumption of normal cell cycle by later time points, in stark contrast to the sustained AA-induced block in DNA synthesis. NZG0809 showed low level of EdU incorporation after AA exposure which was not specifically associated with S-phase.

### 3.4. High Dose Ascorbate Blocks Replication in Both Damaged and Undamaged Cells

**Figure 4:**
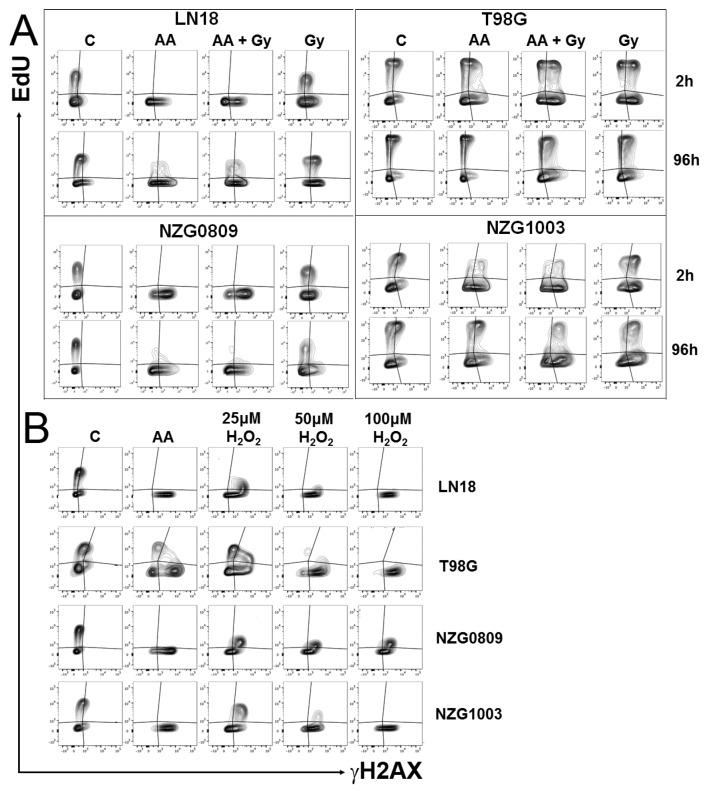
DNA synthesis indicated by EdU incorporation (*y*-axis) versus DNA damage by γH2AX foci (*x*-axis). Cells were treated as indicated and harvested after 2 h (A and B) or 96 h (**A**). (**B**) Effect of a 1 h exposure to 5 mM AA compared with that of different concentrations of H_2_O_2_. Flow cytometry plots are representative of at least three independent experiments.

The loss of DNA synthesis in AA-treated cells was profound and long-lasting with no recovery of affected cell lines over a four day period. However, the DNA damage data indicated that a small proportion of cells did not sustain any DNA damage, neither γH2AX, DSBs nor 8OH-dG. We therefore directly compared DNA damage with DNA synthesis. DNA damage in untreated controls was visible in both replicating and non-replicating cells, as expected from genetically unstable GBMs [21]. Both damaged and undamaged cells continued to synthesize DNA after radiation. However, almost no DNA synthesis was seen after AA or combined treatment, not even in undamaged cells in LN18 and NZG0809. A small amount of DNA synthesis was seen in NZG1003, particularly at later time points in DNA damaged cells. Despite sustaining moderate amounts of DNA damage, DNA synthesis in T98G was relatively unaffected by AA treatment (Figure 4A). Interestingly, a 1 h exposure to 50–100 μM H_2_O_2_ stopped DNA synthesis, even in T98G (Figure 4B).

### 3.5. High Dose Ascorbate Causes Metabolic Stress

**Figure 5:**
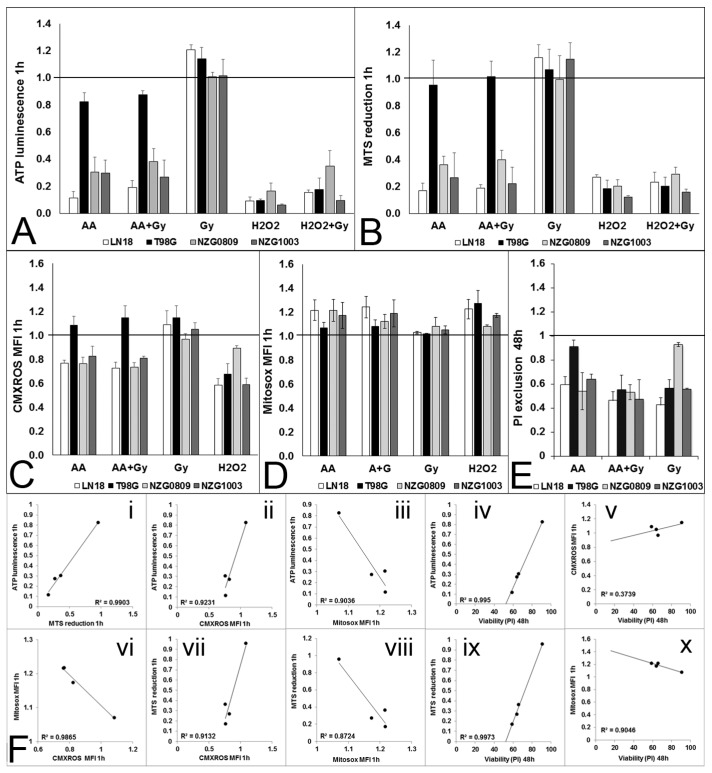
Metabolic stress after 1 h exposure to 5 mM AA, 6 Gy, 5 mM AA + 6 Gy and 500 μM H_2_O_2_ compared to untreated controls (set at 1). (**A**) Cellular ATP levels measured by luminescence; (**B**) Cellular NADH flux measured by MTS reduction; (**C**) Mitochondrial membrane potential measured by CMXROS; (**D**) Mitochondrial superoxide production measured by Mitosox. (**E**) Viability measured as PI exclusion 48 h after treatments. Fold change compared with untreated control cells. ATP luminescence, MTS reduction and CMXRos MFI were all significantly higher after 6 Gy treatment compared with all other treatments for LN18, NZG0809 and NZG1003. Viability after 5 mM AA treatment was significantly higher for T98G and after 6 Gy treatment for NZG0809. (*p* < 0.05: un-paired two-tailed student t-test). Values are averages ± SEM of at least 3 independent experiments in triplicate. (**F**) Correlations between cellular ATP levels, MTS reduction, mitochondrial membrane potential, mitochondrial superoxide production (all at 1 h) and cell viability at 48 h. Each dot represents one of the four cell lines.

Cells with damaged DNA can no longer replicate because of genotoxic stress. However, the observation that replication had also stopped in cells without apparent genotoxic stress was unexpected. It suggests that DNA damage, at least for some cells, is not the primary driver to replication loss, and subsequent cell death. High dose AA has also been shown to decrease ATP levels [3,4,5,6,16]. We also found that ATP levels declined significantly after a 1 h exposure to 5 mM AA in LN18, NZG0809 and NZG1003 but not in T98G. In comparison, 500 μM H_2_O_2_ decreased ATP levels more than 5 mM AA in all cell lines, including T98G (Figure 5A). We next determined the effect of treatments on cellular NADH flux by measuring reduction of the water soluble tetrazolium salt, MTS [30]. We found a substantial decrease in MTS reduction 1 h after AA treatment, combined treatment and after 500 μM H_2_O_2_ in LN18, NZG0809 and NZG1003. MTS reduction in T98G was only minimally affected by 5 mM AA but strongly inhibited by 500 μM H_2_O_2_ (Figure 5B). Robust mitochondrial electron transport (MET) activity results in a strong potential across the inner mitochondrial membrane. We found a decrease in mitochondrial membrane potential in LN18, NZG0809 and NZG1003 1 h after exposure to AA treatments, whereas H_2_O_2_ treatment decreased the mitochondrial membrane potential in all four cell lines (Figure 5C). Interestingly, we found an increase in mitochondrial superoxide levels after exposure to AA and H_2_O_2_ treatments (Figure 5D). Exposure to radiation did not affect ATP levels, MTS reduction, mitochondrial membrane potential or mitochondrial superoxide production in any of the cell lines at such early time points. This was not unexpected as the effects of radiation only become evident at later time points [20]. We previously measured viability 48 h after treatments and found that viability of LN18, NZG0809 and NZG1003 cells decreased substantially after AA treatments. T98G was relatively insensitive to AA whereas NZG1003 was relatively insensitive to radiation (Figure 5E). Correlational analysis (Figure 5F) showed that 1 h after exposure to AA, cellular ATP levels strongly correlated with MTS reduction and mitochondrial membrane potential; MTS reduction strongly correlated with mitochondrial membrane potential; 1 h ATP levels and MTS reduction correlated strongly with 48 h survival.

## 4. Discussion

We previously showed that high dose AA generates γH2AX lesions and causes accumulation of cells in S-phase [23,24]. In this paper, we analysed the extent of genotoxic and metabolic stress and the effect on DNA synthesis by high dose AA, radiation and combined treatments. With respect to genotoxic stress, we verified that high dose AA can indeed generate DSBs (as measured by comet tail assay) as well as 8OH-dG lesions. The increase in 8OH-dG lesions over time suggests effective base excision repair at early time points, but sustained free radical production combined with a lack of effective base excision repair at later time points. This may be caused by low cellular ATP levels. The small amount of EdU incorporation in NZG0809 throughout the cell cycle after AA exposure most likely reflects DNA synthesis as part of base excision repair, or other repair mechanisms, rather than DNA replication [31].

DNA synthesis blockade was expected in cells with DNA damage. However, the fact that undamaged cells were also unable to synthesise DNA in a sustained manner was unexpected and suggests that high dose AA directly affects cell metabolism. AA has been previously reported to decrease ATP levels in neuroblastoma cells [2], prostate cancer [3], ovarian cancer [4] pancreatic cancer [5]. This decrease in ATP was shown to be a result of genotoxic stress in the form of 8OH-dG lesions, which were repaired by PARP-1, leading to consumption of cytoplasmic NAD^+^ (a cofactor of PARP-1). Decreased levels of NAD^+^ inhibited glycolysis and glycolytic ATP production [2,3,4,5]. However, the cells that remained undamaged also stopped synthesising DNA and yet they have no need to activate PARP-1 with subsequent depletion of NAD^+^ levels. We hypothesise that undamaged cells do not synthesise DNA because of depleted cellular ATP, due to direct oxidative damage to components of the energy metabolism pathways. In support of this hypothesis, Yun and colleagues recently showed that intracellular reduction of DHA to AA killed glycolysis-driven KRAS and BRAF mutated colorectal cancer cells through inhibition of glycolysis resulting in ATP depletion [6,7].

Intracellular NADH flux is a good measure of overall cellular energy metabolism. The tetrazolium dye MTS is reduced intracellularly (predominantly by NADH generated during glycolysis) as well as extracellularly (predominantly by NADH originating from the mitochondria) in the presence of an intermediate electron acceptor [30]. The strong decrease in MTS reduction at 1 h closely mimicked the strong drop in cellular ATP levels as well as a decrease in mitochondrial membrane potential. This may reflect both a lack of NAD^+^ (required to fix 8OH-dG lesions) and/or a direct inhibition of glycolysis, Krebs cycle and MET activity, possibly due to oxidative damage to their components. In this respect it is of interest to note that H_2_O_2_ was previously shown to specifically damage the adenine nucleotide translocase (ANT) [32]. ANT is an inner mitochondrial membrane translocase which delivers ATP from the mitochondrial matrix to hexokinase II to facilitate the first step in glycolysis [33]. Oxidative damage to ANT inhibits glycolysis and thus glycolytic ATP production [32]. Declining glycolytic rates limit the amount of pyruvate entering the mitochondria which limits Krebs cycle activity, decreasing NADH and FADH2 levels that fuel MET, oxidative phosphorylation (OXPHOS), mitochondrial membrane potential and generate mitochondrial ATP. Superoxide is produced in the mitochondria during MET as a result of premature leakage of electrons at respiratory complexes I and III [34]. The small increase in superoxide levels we observed, combined with a decrease in membrane potential after AA and H_2_O_2_ treatments, suggests increased leakage due to oxidative damage to respiratory complexes. Both ATP level and MTS reduction 1 h after AA exposure were excellent predictors for cell survival 48 h later. Results presented in this paper show that both damaged and undamaged cells halt DNA synthesis within 2 h of exposure to AA which is not resolved 4 days later. This suggests that high dose AA and H_2_O_2_ generate both genotoxic and metabolic stress which contribute to blocking DNA synthesis in AA sensitive GBM cell lines. The specific contribution of each type of stress is likely to differ between cell lines and between cells of the same cell line.

The effect of H_2_O_2_ and high dose AA as mediators of genotoxic and metabolic stress were very similar in three of the four cell lines—T98G cells were less affected by AA than by H_2_O_2_ in all respects. This was expected, as this cell line is less sensitive to AA due to its high antioxidant capacity with an IC_50_ that is at least two times higher than that of the other cell lines [24]. It is possible that the antioxidant capacity of T98G was overwhelmed by a H_2_O_2_ bolus but able to cope with the H_2_O_2_ generated over a period of time from external AA.

## 5. Conclusions

This paper confirms that the mechanism of action of high dose AA is likely mediated by H_2_O_2_ generation as exposure to high dose AA and H_2_O_2_ abrogated DNA synthesis in cells with damaged and undamaged DNA. Both genotoxic stress and metabolic stress contributed to DNA synthesis arrest in DNA damaged cells. However, DNA synthesis arrest in undamaged cells can only be explained by direct oxidative damage to components of mitochondrial energy production.

## References

[1] Parrow N.L., Leshin J.A., Levine M. (2013). Parenteral ascorbate as a cancer therapeutic: A reassessment based on pharmacokinetics. Antioxid. Redox Signal..

[2] Bruchelt G., Schraufstätter I.U., Niethammer D., Cochrane C. (1991). Ascorbic Acid Enhances the Effects of 6-Hydroxydopamine and H_2_O_2_ on Iron-dependent DNA Strand Breaks and Related Processes in the Neuroblastoma Cell Line SK-N-SH. Cancer Res..

[3] Chen P., Yu J., Chalmers B., Drisko J., Yang J., Li B., Chen Q. (2012). Pharmacological ascorbate induces cytotoxicity in prostate cancer cells through ATP depletion and induction of autophagy. Anticancer Drugs.

[4] Ma Y., Chapman J., Levine M., Polireddy K., Drisko J. (2014). High dose parenteral ascorbate enhanced chemosensitivity of ovarian cancer and reduced toxicity of chemotherapy. Sci. Transl. Med..

[5] Du J., Martin S.M., Levine M., Wagner B.A., Buettner G.R., Wang S.H., Taghiyev A.F., Du C., Knudson C.M., Cullen J.J. (2010). Mechanisms of Ascorbate-Induced Cytotoxicity in Pancreatic Cancer. Clin. Cancer Res..

[6] Yun J., Mullarky E., Lu C., Bosch K., Kavalier A., Rivera K., Roper J., Chio I.I.C., Giannopoulou E.G., Rago C. (2015). Vitamin C selectively kills *KRAS* and *BRAF* mutant colorectal cancer cells by targeting GAPDH. Science.

[7] Van der Reest J., Gottlieb E. (2016). Anti-cancer effects of vitamin C revisited. Cell Res..

[8] Kuiper C., Dachs G.U., Munn D., Currie M.J., Robinson B.A., Pearson J.F., Vissers M.C.M. (2014). Increased Tumor Ascorbate is Associated with Extended Disease-Free Survival and Decreased Hypoxia-Inducible Factor-1 Activation in Human Colorectal Cancer. Front Oncol..

[9] Kuiper C., Molenaar I.G.M., Dachs G.U., Currie M.J., Sykes P.H., Vissers M.C.M. (2010). Low ascorbate levels are associated with increased hypoxia-inducible factor-1 activity and an aggressive tumor phenotype in endometrial cancer. Cancer Res..

[10] Bonuccelli G., De Francesco E.M., DeBoer R., Tanowitz H.B., Lisanti M.P. (2017). NADH autofluorescence, a new metabolic biomarker for cancer stem cells: Identification of Vitamin C and CAPE as natural products targeting “stemness”. Oncotarget.

[11] Du J., Cullen J.J., Buettner G.R. (2012). Ascorbic acid: Chemistry, biology and the treatment of cancer. Buochim. Biophys. Acta.

[12] Du J., Cieslak J.A., Welsh J.L., Sibenaller Z.A., Allen B.G., Wagner B.A., Kalen A.L., Doskey C.M., Strother R.K., Button A.M. (2015). Pharmacological Ascorbate Radiosensitizes Pancreatic Cancer. Cancer Res..

[13] Chen Q., Espey M.G., Sun A.Y., Pooput C., Kirk K.L., Krishna M.C., Khosh D.B., Drisko J., Levine M. (2008). Pharmacologic doses of ascorbate act as a prooxidant and decrease growth of aggressive tumor xenografts in mice. Proc. Natl. Acad. Sci. USA.

[14] Monti D.A., Mitchell E., Bazzan A.J., Littman S., Zabrecky G., Yeo C.J., Pillai M.V., Newberg A.B., Deshmukh S., Levine M. (2012). Phase I evaluation of intravenous ascorbic acid in combination with gemcitabine and erlotinib in patients with metastatic pancreatic cancer. PLoS ONE.

[15] Welsh J.L., Wagner B.A., van’t Erve T.J., Zehr P.S., Berg D.J., Halfdanarson T.R., Yee N.S., Bodeker K.L., Du J., Roberts L.J. (2013). Pharmacological ascorbate with gemcitabine for the control of metastatic and node-positive pancreatic cancer (PACMAN): results from a phase I clinical trial. Cancer Chemother. Pharmacol..

[16] Deubzer B., Mayet F., Kuçi Z., Niewisch M., Merkel G., Handgretinger R. (2010). H_2_O_2_-mediated cytotoxicity of pharmacologic ascorbate concentrations to neuroblastoma cells: potential role of lactate and ferritin. Cell. Physiol. Biochem..

[17] Duarte T.L., Almeida G.M., Jones G.D.D. (2007). Investigation of the role of extracellular H_2_O_2_ and transition metal ions in the genotoxic action of ascorbic acid in cell culture models. Toxicol. Lett..

[18] Valko M., Rhodes C.J., Moncol J., Izakovic M., Mazur M. (2006). Free radicals, metals and antioxidants in oxidative stress-induced cancer. Chem. Biol. Interact..

[19] Baader S.L., Bruchelt G., Carmine T.C., Lode H.N., Rieth A.G., Niethammer D. (1994). Ascorbic acid mediated iron release from cellular ferritin and its relation to DNA strand break formation in neuroblastoma cells. J. Cancer Res. Clin. Oncol..

[20] Wouters B., Begg A., Joiner M.C., van der Kogel A.J. (2009). Irradiation-induced damage and the DNA damage response. Basic Clinical Radiobiology.

[21] Bartkova J., Hamerlik P., Stockhausen M.-T., Ehrmann J., Hlobilkova A., Laursen H., Kalita O., Kolar Z., Poulsen H.S., Broholm H. (2010). Replication stress and oxidative damage contribute to aberrant constitutive activation of DNA damage signalling in human gliomas. Oncogene.

[22] Stupp R., Hegi M., Mason W., van den Bent M., Taphoorn M., Janzer R., Ludwin S.K., Allgeier A., Fisher B., Belanger K. (2009). Effects of radiotherapy with concomitant and adjuvant temozolomide versus radiotherapy alone on survival in glioblastoma in a randomised phase III study: 5-year analysis of the EORTC-NCIC trial. Lancet Oncol..

[23] Herst P.M., Broadley K.W.R., Harper J.L., McConnell M.J. (2012). Pharmacological concentrations of ascorbate radiosensitize glioblastoma multiforme primary cells by increasing oxidative DNA damage and inhibiting G2/M arrest. Free Radic. Biol. Med..

[24] Castro M., McConnell M., Herst P. (2014). Radio-sensitisation by pharmacological ascorbate in glioblastoma multiforme cells, human glial cells and HUVECs depends on their antioxidant and DNA repair capabilities and is not cancer specific. Free Radic. Biol. Med..

[25] Katsube T., Mori M., Tsuji H., Shiomi T., Wang B., Liu Q., Nenoi M., Onoda M. (2014). Most hydrogen peroxide-induced histone H2AX phosphorylation is mediated by ATR and is not dependent on DNA double-strand breaks. J. Biochem..

[26] Berniak K., Rybak P., Bernas T., Zarębski M., Biela E., Zhao H., Darzynkiewicz Z., Dobrucki J.W. (2013). Relationship between DNA damage response, initiated by camptothecin or oxidative stress, and DNA replication, analyzed by quantitative 3D image analysis. Cytom. A.

[27] Zhao H., Dobrucki J., Rybak P., Traganos F., Halicka D.H., Darzynkiewicz Z. (2011). Induction of DNA damage signaling by oxidative stress in relation to DNA replication as detected using “click chemistry”. Cytom. A.

[28] De Feraudy S., Revet I., Bezrookove V., Feeney L., Cleaver J.E. (2010). A minority of foci or pan-nuclear apoptotic staining of gammaH2AX in the S phase after UV damage contain DNA double-strand breaks. Proc. Natl. Acad. Sci. USA.

[29] Broadley K.W.R., Hunn M.K., Farrand K.J., Price K.M., Grasso C., Miller R.J., Hermans I.F., McConnell M.J. (2011). Side population is not necessary or sufficient for a cancer stem cell phenotype in gliobastoma multiforme. Stem Cells.

[30] Berrdige M., Herst P., Tan A. (2005). Tetrazolium dyes as tools in cell biology: New insights into their cellular reduction. Biotechnol. Annu. Rev..

[31] Limsirichaikul S., Niimi A., Fawcett H., Lehmann A., Yamashita S., Ogi T. (2009). A rapid non-radioactive technique for measurement of repair synthesis in primary human fibroblasts by incorporation of ethynyl deoxyuridine (EdU). Nucleic Acids Res..

[32] Yan L.J., Sohal R.S. (1998). Mitochondrial adenine nucleotide translocase is modified oxidatively during aging. Proc. Natl. Acad. Sci. USA.

[33] Cerella C., Dicato M., Diederich M. (2014). Modulatory roles of glycolytic enzymes in cell death. Biochem. Pharmacol..

[34] Quinlan C.L., Perevoshchikova I.V., Hey-Mogensen M., Orr A.L., Brand M.D. (2013). Sites of reactive oxygen species generation by mitochondria oxidizing different substrates. Redox Biol..

